# Finerenone Alleviates Over-Activation of Complement C5a-C5aR1 Axis of Macrophages by Regulating G Protein Subunit Alpha i2 to Improve Diabetic Nephropathy

**DOI:** 10.3390/cells14050337

**Published:** 2025-02-26

**Authors:** Zi-Han Li, Zi-Jun Sun, Sydney C. W. Tang, Ming-Hui Zhao, Min Chen, Dong-Yuan Chang

**Affiliations:** 1 Renal Division, Department of Medicine, Peking University First Hospital, Beijing 100034, China; lizihan@bjmu.edu.cn (Z.-H.L.); zjsun@email.sdfmu.edu.cn (Z.-J.S.); mhzhao@bjmu.edu.cn (M.-H.Z.); 2 Peking University Institute of Nephrology, Beijing 100034, China; 3 Key Laboratory of Renal Disease, Ministry of Health of China, Beijing 100034, China; 4 Key Laboratory of Chronic Kidney Disease Prevention and Treatment, Peking University, Ministry of Education, Beijing 100034, China; 5 Department of Nephrology, Peking University First Hospital, State Key Laboratory of Vascular Homeostasis and Remodeling, Peking University, Beijing 100034, China; 6 Research Units of Diagnosis and Treatment of Immune-Mediated Kidney Diseases, Chinese Academy of Medical Sciences, Beijing 100730, China; 7 Division of Nephrology, Department of Medicine, School of Clinical Medicine, The University of Hong Kong, Queen Mary Hospital, Hong Kong 999077, China; scwtang@hku.hk

**Keywords:** diabetic nephropathy, finerenone, MR, C5aR1, Gnαi2

## Abstract

Diabetic nephropathy (DN), one of the most common complications of diabetes mellitus (DM), accounts for a major cause of chronic kidney disease (CKD) worldwide, with a complicated pathogenesis and limited effective strategies nowadays. The mineralocorticoid receptor (MR) is a classical ligand-activated nuclear transcription factor. It is expressed in the renal intrinsic and immune cells, especially macrophages. Over-activation of the MR was observed in patients with DN and was associated with DN prognosis. The renoprotective role of a new generation of non-steroidal selective mineralocorticoid receptor antagonist (MRA), finerenone, has been confirmed in DM and CKD patients. However, the mechanism by which finerenone improves renal inflammation in DN has yet to be completely understood. It was found in this research that the oral administration of finerenone attenuated the kidney injuries in established DN in *db*/*db* mice, and particularly improved the pathological changes in the renal tubulointerstitia. Specifically, finerenone inhibited the over-activation of the MR in macrophages, thereby reducing the expression of G protein subunit alpha i2 (*GNAI2*, Gnαi2), a key downstream component of the C5aR1 pathway. Animal experiments demonstrated that *C5aR1* knockout alleviated renal injuries, confirming the critical pathogenic role of C5aR1 in DN. Moreover, finerenone mitigated inflammatory and chemotaxis responses by downregulating Gnαi2 in macrophages. These effects were reflected by reduced expressions of the pro-inflammatory chemokines CXCL15 and CCL2, the regulation of macrophage polarization and improvements in apoptosis. This study intends to understand the protective role of finerenone in DN, which is conducive to revealing the pathophysiological mechanism of DN and further optimizing the treatment of DN patients.

## 1. Introduction

The total number of adults with diabetes mellitus (DM) in China is about 180 million, ranking first in the world [1]. Presently, strategies for the prevention and treatment of DN mainly focus on controlling the risk factors, including blood glucose, blood pressure and hyperlipidemia [2,3]. Although these strategies explicitly improve the prognosis of DN, the treatment strategies are still relatively limited, and the prognosis of DN is of concern. Aldosterone is an essential part of the mineralocorticoid hormone secreted by the spherical band cells of the adrenal cortex [4]. Aldosterone can be regulated by many factors, including renin–angiotensin, adrenocorticotropin, Na^+^ and K^+^ levels. Aldosterone binds to the mineralocorticoid receptor (MR) to enhance Na^+^-K^+^ exchange mainly through its role as a ligand-activated nuclear transcription factor [5]. It improves the membrane permeability and renal tubule reabsorption of Na^+^, resulting in water reabsorption increase and salt metabolism balance [6]. Mineralocorticoid receptors are widely expressed in immune cells, especially macrophages, in addition to the intrinsic cells of kidney and heart tissues [7,8]. The over-activation of the MR in patients with DN causes metabolic disorders, including hypokalemia and sodium and water retention, and mediates the onset and progression of tissue inflammation and fibrosis of kidneys and hearts, which further leads to a variety of adverse renal and cardiac outcomes [9]. The FIDELIO-DKD study [10] and the FIGARO-DKD study [11] have confirmed finerenone, a new generation of MRA, and its renoprotective role in diabetic patients with CKD. Therefore, finerenone could strongly inhibit the over-activation of the MR in kidney tissues, thereby inhibiting and delaying the progression of inflammatory fibrosis and playing a direct role in kidney protection, as reported.

The over-activation of the complement system is a key contributor to the pathogenesis of DN [12,13]. C5a, one of the classic allergic toxins, is a crucial molecule in activating the complement system [14]. There are two C5a receptors, termed C5aR1 (also known as CD88) and C5aR2 (also known as GPR77 or C5L2) [15]. C5aR1, which has seven transmembrane domains, is considered the main receptor in inflammation and chemotaxis [16]. C5aR1 is mainly expressed in mononuclear macrophages and renal tubular epithelial cells in kidneys in DN [17,18]. C5a plays an important role in inflammatory responses by binding to C5aR1, secreting cytokines and chemokines, recruiting immune cells and activating downstream signaling pathways [19]. C5aR1 is a typical G protein-coupled receptor (GPCR) [20,21]. The GPCRs are general terms for a large class of membrane protein receptors, and their downstream signaling pathways are varied depending on the unique G protein subunits [22,23]. Previous studies have confirmed that the chemotaxis generated by activating the C5a-C5aR1 axis in macrophages participated in the pathogenesis of various inflammatory diseases [24,25,26]. It was reported that the key G protein subunit activated by C5a-C5aR1 was Gnαi2 in macrophages, and C5a exerted its powerful chemotaxis after binding with C5aR1 containing this subunit [27].

In this study, we found that finerenone inhibited the transcription of the *Gnαi2* gene by antagonizing the MRs in renal macrophages in DN. The downregulation of Gnαi2 in renal macrophages could alleviate the over-activation of the C5a-C5aR1 axis and its downstream inflammatory damages, thus ultimately exerting a renoprotective role in DN.

## 2. Materials and Methods

### 2.1. Human Renal Biopsy Samples

The renal biopsies were obtained during routine clinical diagnosis. Thirty diabetes patients with biopsy-proven DN were enrolled. Ten samples of healthy controls were collected from individuals undergoing tumor nephrectomies who had no history of diabetes or kidney disease. The investigation adhered to the principles of the Declaration of Helsinki and received approval from the Ethics Committee (No. 2024-252; Approval Date: 27 January 2025).

### 2.2. Animal Model

The animal studies were performed under protocols sanctioned by the Laboratory Animal Ethics Committee of Peking University First Hospital (Ethic Approval No. J2024127; Approval Date: 28 October 2024). The studies utilized 6-week-old male diabetic *db*/*db* mice (C57BL/6J-LepR*^db^*^/*db*^) alongside their non-diabetic *db*/*m* (C57BL/6J-LepR*^db^*^/*+*^) littermates, obtained from GemPharmatech (Nanjing, China). These specimens were maintained at Peking University First Hospital’s facility under specific pathogen-free (SPF) conditions, with controlled environmental parameters including alternating 12 h periods of light and darkness and an ambient temperature regulated between 24 and 26 °C. The *db*/*db* mice were split randomly into two experimental cohorts, with one receiving finerenone treatment and another serving as a vehicle treatment group. Additionally, the *db*/*m* mice were utilized as the normal control group. The intervention protocol involved daily oral administration of finerenone (BAY 94-8862, Leverkusen, Germany) at 10 mg/kg/day to the treatment group, commencing at eight weeks and continuing for a duration of ten weeks. The vehicle treatment and normal control groups received 0.2 mL solvent through oral gavage daily over a 10-week period.

### 2.3. HFD/STZ-Induced DN Model in Global C5aR1 Knockout Mice

Male littermate wildtype and *C5aR1* KO (*C5aR1*^−/−^) C57BL/6J background mice were purchased from GemPharmatech (Nanjing, China). DN was established in the mice model through a combination of high-fat diet (HFD) feeding and streptozotocin (STZ) administration. To be specific, the mice were fed with the HFD (60% fat, HfkBio, Beijing, China) for 1 month. After starving for 4–6 h, intraperitoneal injections of 60 mg/kg STZ (Sigma-Aldrich, St. Louis, MO, USA) dissolved in 50 mM sodium citrate buffer (PH 4.5) were performed for 5 continuous days. Then, the HFD feeding style was maintained for 20 weeks. Two weeks after the final STZ injection, mice were considered successful with a random blood glucose >16.7 mmol/L. The mice fed with the SFD diet (10% fat) and injected with vehicle were considered as the non-diabetic controls. Weekly assessments including measurements of blood glucose levels and body weight, complemented by biochemical evaluations of blood and full-day urine samples, were collected every 2 to 4 weeks. For morphological assessment, mice kidney specimens were gathered at the experimental endpoint. All animals were housed in Peking University First Hospital under the environmental conditions mentioned above.

### 2.4. Cell Culture

RAW 264.7, a mouse macrophage cell line, was obtained from Ubigene Biosciences (Guangzhou, China). The cell culture was maintained at 37 °C in a humidified atmosphere containing 5% CO_2_. The culture medium consisted of DMEM supplemented with 10% fetal bovine serum and 1% penicillin–streptomycin solution (Gibco, Grand Island, NY, USA). Upon reaching 70–80% confluency, the cells underwent treatment with 30 mmol/L high glucose and finerenone (5 mM; MedChemExpress, Monmouth Junction, NJ, USA) [28] for 72 h.

### 2.5. Biochemical Analysis of Serum/Urine/Cell Supernatant Samples

Using electronic balances, the mice were weighed with a precision of 0.1 g. Following the administration of avertin anesthesia, blood samples were obtained via angular vein collection. The blood samples were analyzed to measure key metabolic indicators in the mice, including the glucose levels after fasting, as well as the serum lipid parameters (total cholesterol and triglycerides) using standardized test kits. To assess renal function, urine samples were obtained over a 24 h period utilizing metabolic cage systems. The albumin content in the urine specimens was quantified through mouse-specific ELISA testing (Bethyl Laboratories, Montgomery, TX, USA), while the urinary creatinine was assessed using DICT-500 assay kit (BioAssay Systems, Hayward, CA, USA). The albumin-to-creatinine ratio (ACR) was calculated by normalizing the urinary albumin concentrations against the creatinine levels. The levels of the chemokines CXCL15 and CCL2 in the mice urine or cell supernatant were measured by commercial ELISA kits (FineTest, Wuhan, China).

### 2.6. Renal Histology

The excised mouse kidneys were immersed in 4% paraformaldehyde (PFA) solution and maintained overnight for fixation. The tissue specimens were embedded in paraffin and sectioned at 2 μm thickness, followed by Periodic acid-Schiff (PAS) staining using BA-4080B reagent from BASO (Zhuhai, China). For the assessment of glomerular and tubulointerstitial damage, multiple fields were analyzed—a minimum of 10 glomeruli or microscopic views per section were captured digitally at 400× magnification. The analysis was performed using Image-Pro Plus V.6.0 software, Media Cybernetics, Rockville, MD, USA. The extent of the mesangial matrix expansion was calculated as a percentage within the individual glomeruli [29]. The assessment of interstitial fibrosis and tubular atrophy (IFTA) involved examining tubular dilation patterns, cellular atrophy and epithelial cell loss. The evaluation was conducted across 20 distinct fields for each kidney section as follows; 0: no injury, 1: <25%, 2: 25–50%, 3: 50–75%, 4: >75% of the proportion with tubulointerstitial injury [30,31]. The histological analysis was performed in a blinded fashion.

### 2.7. Transmission Electron Microscopy

The kidney cortex specimens were sectioned into three portions and fixed immediately using 3% glutaraldehyde solution. The subsequent processing steps were completed at the Electron Microscopy Facility of Peking University First Hospital. From each mouse, three glomeruli were randomly selected and processed into ultrathin sections. Using a transmission electron microscope (Tecnai G^2^ 20 TWIN; Thermo Fisher Scientific, Waltham, MA, USA), ten non-overlapping representative digital images were captured from each glomerular sample. Image J software (64-bit Java version 1.6.0, National Institutes of Health, Bethesda, MD, USA) was employed to measure and analyze the thickness of the glomerular basement membrane (GBM) and the width of foot processes (FPW) [32].

### 2.8. Tissue RNA Isolation and RT-qPCR

The mouse kidney tissues and cell lines underwent RNA isolation using RNAprep pure Kits from Tiangen Biotech (Beijing, China). The specific primers employed for mRNA detection are shown in Table 1. We measured the RNA content using a NanoDrop 1000 spectrophotometer (Thermo Fisher Scientific, Waltham, MA, USA). Subsequently, the RNA samples were reverse transcribed to cDNA utilizing High-Capacity cDNA Reverse Transcription Kits (Applied Biosystems, Foster City, CA, USA) following the manufacturer’s protocols. For quantitative analysis, we conducted RT-qPCR experiments on a LightCycler480 platform (Roche, Basel, Switzerland) with SYBR Green Master Mix (Thermo Fisher Scientific, Waltham, MA, USA). The gene expression levels were standardized against *18 s* rRNA in the mouse samples and calculated relative to the control conditions.

### 2.9. Western Blot

To extract the total protein content, the cells were lysed using RIPA lysis buffer. The protein samples underwent electrophoretic separation on sodium dodecyl sulfate (SDS) polyacrylamide gels, followed by being transferred onto PVDF membranes. After blocking with 5% milk solution at room temperature for 60 min, the membranes were probed with specific antibodies targeting Gnαi2 (1:1000; 11136-1-AP, Proteintech, Wuhan, China) and actin (1:1000; ab8226, Abcam, Waltham, MA, USA) at 4 °C overnight. The following day, the corresponding secondary antibodies were applied for an hour at room temperature. Protein bands were detected using an ECL Plus detection kit (GE Healthcare, Chicago, IL, USA) and visualized through autoradiography.

### 2.10. Immunofluorescence and Immunohistochemistry

The tissue and cell immunofluorescence staining was performed by incubating several primary antibodies against C5aR1 (1:200; ab117579, Abcam, Waltham, MA, USA), Gnαi2 (1:200; 11136-1-AP, Proteintech, Wuhan, China) and CD68 (1:100; ab955, Abcam, Waltham, MA, USA). Following this step, the samples were treated with Alexa 488/555-linked secondary antibodies (Invitrogen, Carlsbad, CA, USA). For nuclear visualization, DAPI staining was performed using reagents from ZSGB-BIO (Beijing, China). The paraffin-embedded kidney sections (4 µm) were stained with primary antibodies against C3a (1:150; ab36385, Abcam, Waltham, MA, USA), C5a (1:300; ab11878, Abcam, Waltham, MA, USA), C5aR1 (1:500; ab117579, Abcam, Waltham, MA, USA) and Gnαi2 (1:500; 11136-1-AP, Proteintech, Wuhan, China). The tissue samples were treated with peroxidase-linked secondary antibodies and kept at 4 °C overnight following the manufacturer’s protocols (ZSGB-BIO, Beijing, China). The stained specimens were photographed using a high-resolution imaging system.

### 2.11. Flow Cytometry

Cell apoptosis was detected by Annexin V/PI commercial kits (Beyotime, Shanghai, China) according to the manufacturer’s instructions. Cluster of differentiation 86 (CD86) and cluster of differentiation 206 (CD206) were utilized as specific markers to distinguish between the M1 and M2 macrophage phenotypes, respectively (Invitrogen, Carlsbad, CA, USA). The expressions of the determined markers were then assessed by flow cytometry.

### 2.12. Bioinformatic Analyses

The three-dimensional structural data of the MR (NR3C2) molecules were retrieved from the Protein Data Bank (PDB) database. To identify potential transcription factor MR binding targets, we performed an analysis using the Cistrome database. *Homo sapiens*, kidney and NR3C2 were chosen in “Species”, “Biological Sources” and “Factors”, respectively. The core targets were subjected to KEGG pathway analyses to explore their biological functions. The pathways showing the highest statistical significance were selected and illustrated according to their “*p*-Value”. For the bioinformatics portion of this study, we employed analytical tools provided by OECloud (https://cloud.oebiotech.com, accessed on 3 May 2024). The signaling pathways of the complement system were obtained from https://www.wikipathways.org/instance/WP2806 (accessed on 4 May 2024).

### 2.13. Statistics Analyses

Data analysis was conducted with GraphPad Prism version 8.4.2, San Diego, CA, USA. Each experiment included a minimum of three independent biological replicates. For the data following a normal distribution, the results were presented as mean values ± standard deviation. The non-normally distributed data were expressed using medians with interquartile ranges. For comparisons between two independent groups, we employed an unpaired two-tailed Student’s *t*-test (for the normally distributed data) or a Mann–Whitney U test (for the non-normally distributed data). When comparing multiple groups, a one-way analysis of variance (ANOVA) was applied, followed by Bonferroni’s post hoc test. Differences were considered significant when the *p*-values were less than 0.05.

## 3. Results

### 3.1. Finerenone Alleviated Renal Injury in db/db Mice

**Figure 1:**
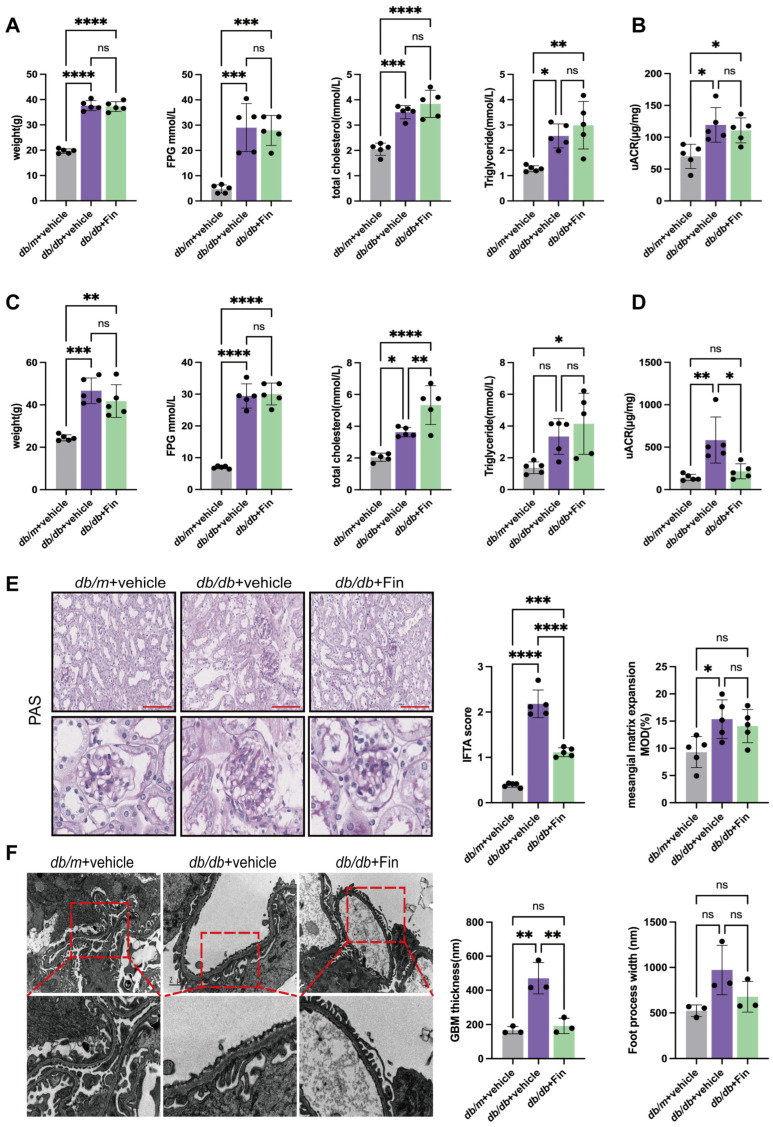
Finerenone alleviated renal injury in *db*/*db* mice. (**A**) Baseline body weight, fast plasma glucose (FPG), serum total cholesterol (TCHO) and triglycerides (TGs) of mice in three groups (*n* = 5 per group). (**B**) Baseline urine albumin-to-creatine ratio (uACR) of mice in three groups (*n* = 5 per group). (**C**) Body weight, FPG, serum TCHO and TGs of mice in three groups upon 10 weeks of finerenone treatment (*n* = 5 per group). (**D**) Urine albumin-to-creatine ratio (uACR) of mice in three groups upon 10 weeks of finerenone treatment (*n* = 5 per group). (**E**) Representative images of morphological examinations by Periodic acid-Schiff (PAS), quantitative analysis of IFTA score and degree of mesangial matrix expansion (*n* = 5 per group). Scale bar: 50 μm. (**F**) Representative images of TEM in mice kidneys and quantitative analysis showing GBM thickness and FPW (*n* = 3 per group). Scale bar: 2 μm. asterisks Statistical significance levels are denoted as follows: * *p* < 0.05, ** *p* < 0.01, *** *p* < 0.001, **** *p* < 0.0001. The term ‘ns’ indicates a non-significant difference (*p* ≥ 0.05).

To evaluate the renal improvement of finerenone in DN mice, the oral administration of finerenone was conducted as mentioned above. In contrast to the *db*/*m* mice, all *db*/*db* mice started to manifest obesity, hyperglycemia, hyperlipidemia and proteinuria at seven weeks of age (Figure 1A,B). Finerenone was administered once daily for ten continuous weeks at the age of eight weeks, and there were no significant improvements in weight loss, FPG, serum triglyceride or total cholesterol levels after finerenone treatment (Figure 1C). Importantly, upon finerenone treatment, the uACR of the mice was significantly lower than that of the vehicle treatment group (215.70 ± 88.73 vs. 583.70 ± 272.30 μg/mg, *p* = 0.0115) (Figure 1D).

Inflammatory cell infiltration in the renal tubulointerstitium and glomerular mesangial matrix expansion, which are typical renal pathological changes in DN, were evaluated by PAS staining. The IFTA scores and the degree of the mesangial matrix expansion were markedly higher in the vehicle treatment group compared with the normal control group. The finerenone treatment group exhibited notable enhancements in IFTA scores when compared with the vehicle treatment group. Nevertheless, the analysis of the mesangial matrix expansion showed no substantial alterations following finerenone administration (Figure 1E). Transmission electron microscopy (TEM) analysis showed that compared with the normal control group, the mean width of the glomerular basement membrane (GBM) was increased in the vehicle treatment group, while it was decreased upon finerenone treatment (470.30 ± 92.08 vs. 166.8 ± 21.30 nm, *p* = 0.0020 and 191.80 ± 44.50 vs. 470.30 ± 92.08 nm, *p* = 0.0032, respectively). However, a similar trend in the foot process width was detected but did not reach a statistical difference (Figure 1F).

### 3.2. Finerenone Affected the C5aR1/Gnαi2 Axis by Inhibiting the Transcriptional Regulation of Gnαi2 by the MR

**Figure 2:**
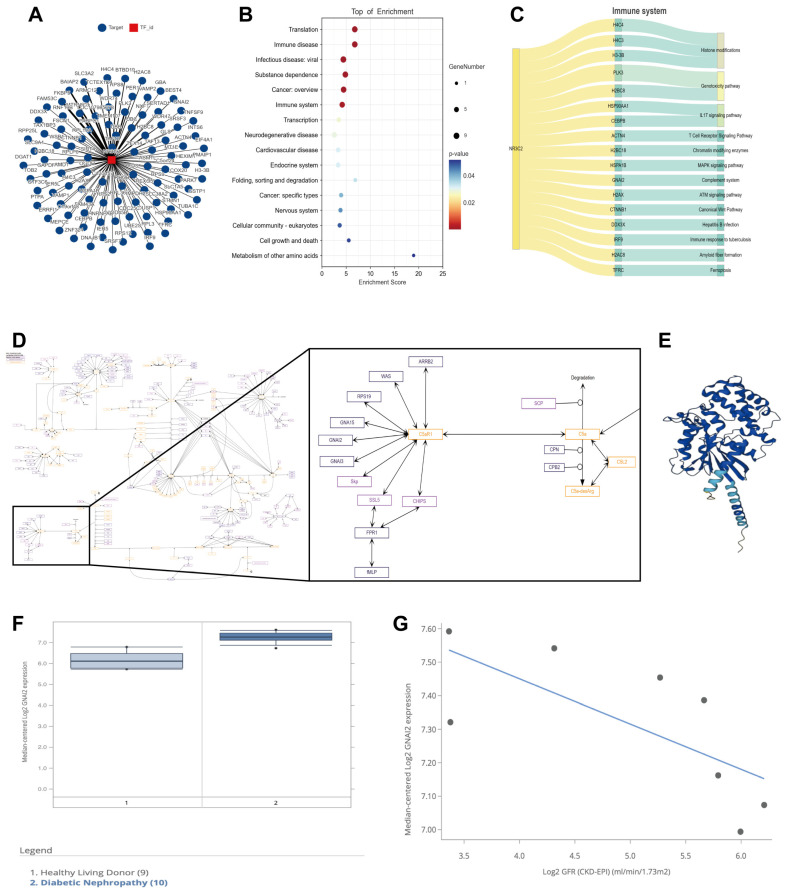
Finerenone affected C5aR1/Gnαi2 axis by inhibiting transcriptional regulation of Gnαi2 by MR. (**A**) All target transcription factors (TFs) regulated by MR were found in human kidneys in Cistrome database. (**B**) Top KEGG enrichment pathways. (**C**) 17 TFs were enriched in immune system. (**D**) C5a-C5aR1-associated signaling pathways of complement system. (**E**) Visualization of 3D structure of Gnαi2. (**F**) mRNA levels of *GNAI2* in renal tubulointerstitia of patients with DN (*n* = 10) and controls (*n* = 9) from ERCB Nephrotic Syndrome TubInt cohort. (**G**) Correlation between tubulointerstitial *GNAI2* mRNA levels and GFRs in DN patients from ERCB Nephrotic Syndrome TubInt dataset (*n* = 8).

To further explore the mechanism by which finerenone alleviated kidney injury in DN using *db*/*db* mice models, we studied the target of finerenone, the MR (NR3C2), and its possible role as a transcription factor. The Cistrome database was used to find putative targets for the transcription factor MR. *Homo sapiens*, kidney and NR3C2 were chosen in “Species”, “Biological Sources” and “Factors”, respectively. A total of 100 putative targets that were regulated by the MR were found in human kidneys. All targets are shown in Figure 2A by a network interaction diagram. A top KEGG enrichment analysis of the 100 putative targets was conducted, and a bubble diagram is constructed in Figure 2B, suggesting that targets were enriched into several categories including the immune system. We found that 17 targets were enriched into the immune system, among which *GNAI2* was involved in the complement system (Figure 2C). The signaling pathways of the complement system, especially those related to the C5a-C5aR1 axis, are shown in Figure 2D. Gnαi2 (encoded by *GNAI2* gene) is a downstream subunit of the G proteins of C5aR1, and its 3D structure is shown in Figure 2E. As a key G protein subunit activated by C5a-C5aR1, it was reported to play an important role in C5a-C5aR1-associated chemotaxis and inflammation in macrophages [27], which was thought to be involved in the development and progression of DN [33,34,35]. The publicly available Nephroseq database (http://v5.nephroseq.org, accessed on 19 February 2025) was utilized for further analysis. In the ERCB Nephrotic Syndrome TubInt database, we found that *GNAI2* mRNA levels were significantly elevated (*p* = 5.41 × 10^−5^) in the renal tubulointerstitia of DN patients (*n* = 10) compared with health controls (*n* = 9) (Figure 2F), and there was a significantly negative correlation between the tubulointerstitial *GNAI2* mRNA levels and glomerular filtration rates (GFRs) in DN patients (*n* = 8, *p* = 0.048, r = −0.712) (Figure 2G).

### 3.3. Finerenone Alleviated C5a-C5aR1-Associated Chemotaxis in db/db Mice

**Figure 3:**
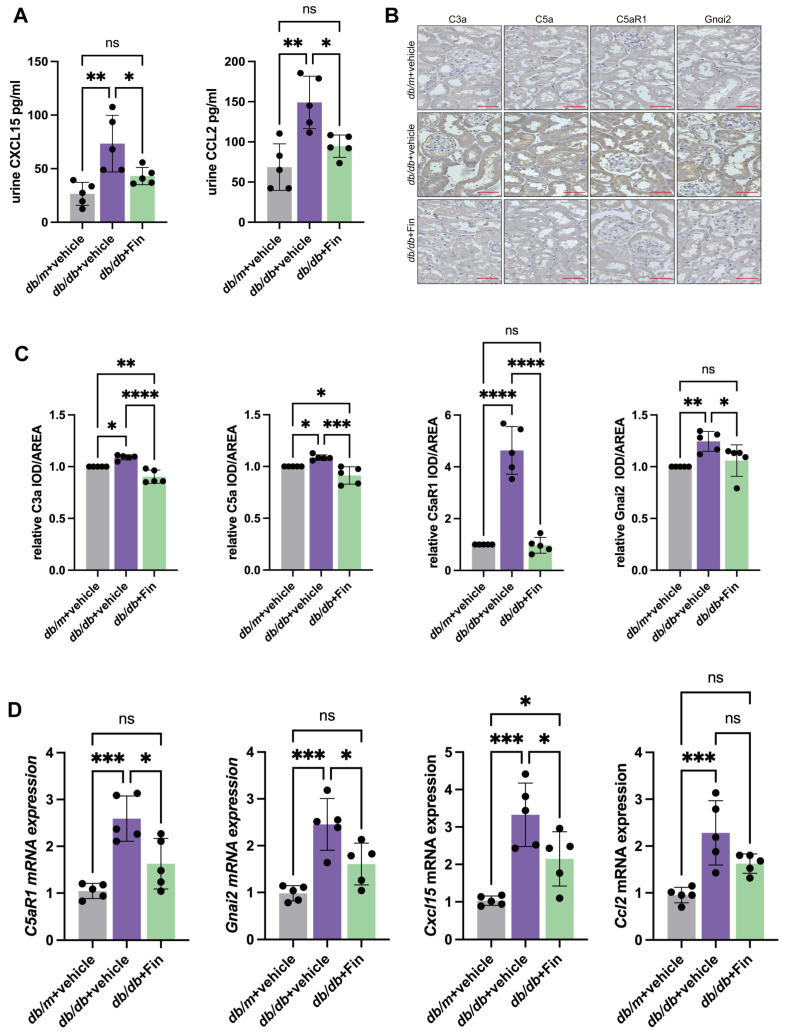
Finerenone alleviated C5a-C5aR1-associated chemotaxis in *db*/*db* mice. (**A**) Levels of CXCL15 and CCL2 in urine of mice in three groups upon 10 weeks of finerenone treatment (*n* = 5 per group). (**B**) Representative IHC images of C3a, C5a, C5aR1 and Gnαi2 in kidney tissues of three groups. Scale bar: 50 μm. (**C**) Quantifications of IHC of C3a, C5a, C5aR1 and Gnαi2 (*n* = 5 per group). (**D**) mRNA levels of *C5aR1*, *Gnαi2*, *Cxcl15* and *Ccl2* in mice kidneys in three groups (*n* = 5 per group). asterisks Statistical significance levels are denoted as follows: * *p* < 0.05, ** *p* < 0.01, *** *p* < 0.001, **** *p* < 0.0001. The term ‘ns’ indicates a non-significant difference (*p* ≥ 0.05).

The ELISA analysis revealed that the mice receiving Finerenone showed markedly reduced urinary CXCL15 and CCL2 concentrations compared with the vehicle treatment group (Figure 3A). In addition, immunohistochemical staining found that kidney C3a, C5a, C5aR1 and Gnαi2 were significantly upregulated in the kidneys of the vehicle treatment group compared with the *db*/*m* mice, which were downregulated by finerenone treatment (Figure 3B,C). Also, we found that the mRNA levels of *C5aR1* and *Gnαi2* and the chemokine *Cxcl15* in the mice kidney tissues were significantly suppressed after finerenone intervention by qPCR analysis (Figure 3D).

### 3.4. C5aR1 Deficiency Alleviated Renal Injury in HFD/STZ-Induced Diabetic Mice

As mentioned above, complement system overactivity has been identified as a key contributor to DN pathological processes. It was reported that DN patients with complement deposition in kidneys by renal immunofluorescence pathology exhibited more serious kidney injury compared to cases without complement deposits [36,37]. The levels of multiple active components of the complement system in the plasma and urine of DN patients were significantly higher than those of diabetic patients without renal involvement. In addition, the levels of active components of the complement system, especially C5a, in urine showed strong associations with the degree of kidney damage in DN [38]. As the main receptor of C5a, C5aR1 was a key regulatory link in renal immune damages induced by the over-activation of the complement system in DN [12].

**Figure 4:**
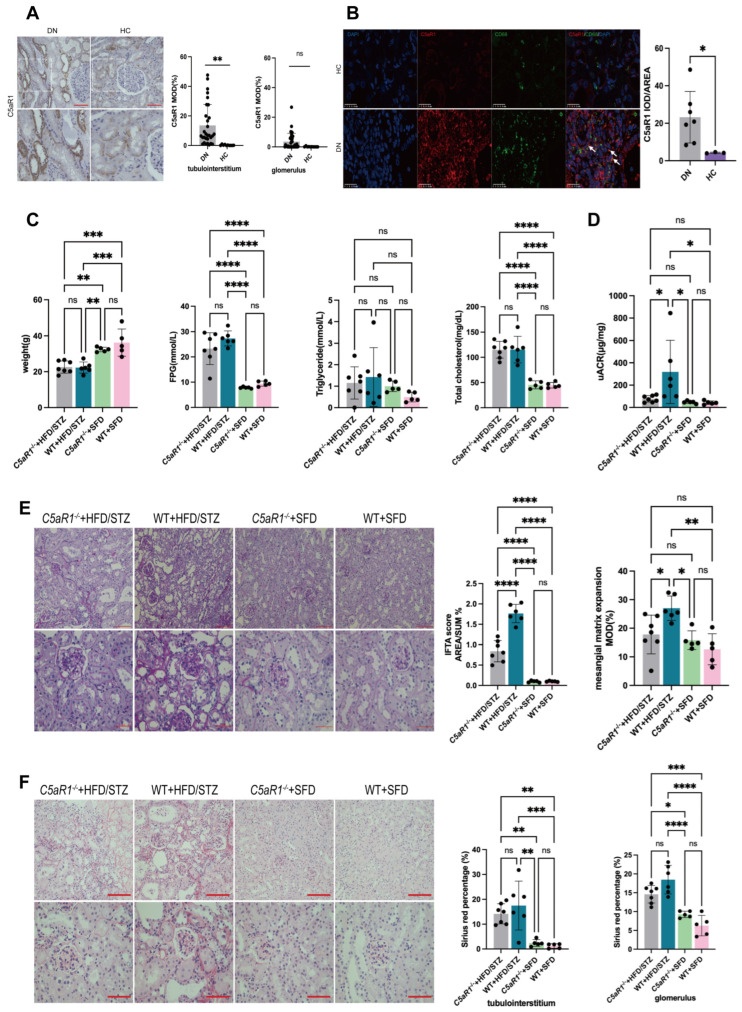
C5aR1 deficiency alleviated renal injury in HFD/STZ-induced diabetic mice. (**A**) Representative IHC images and quantitative analysis of C5aR1 in kidney tissues of DN patients and healthy controls (*n* = 30, 10 respectively). Scale bar: 50 μm. (**B**) Representative IF images of CD68 (green), C5aR1 (red) and nuclear (DAPI) in kidneys from healthy controls and DN patients. Orange-yellow areas indicated by white arrows represent the representative colocalized parts. Scale bar: 50 μm. Quantitative analysis of C5aR1 expressions in two groups (*n* = 7, 3 respectively). (**C**) Body weight, FPG, serum TG and TCHO of mice in four groups (*n* = 7, 6, 5, 5 respectively). (**D**) uACR of mice in four groups (*n* = 7, 6, 5, 5 respectively). (**E**) Representative images of morphological examinations by PAS and quantitative analysis of IFTA scores and degrees of mesangial matrix expansion in four groups (*n* = 7, 6, 5, 5 respectively). Scale bar: 50 μm. (**F**) Representative images and quantitative analysis of Sirius red staining in four groups (*n* = 7, 6, 5, 5 respectively). Scale bar: 50 μm. Asterisks Statistical significance levels are denoted as follows: * *p* < 0.05, ** *p* < 0.01, *** *p* < 0.001, **** *p* < 0.0001. The term ‘ns’ indicates a non-significant difference (*p* ≥ 0.05).

We investigated the expression of C5aR1 in kidney specimens of DN patients. Immunohistochemical staining found that the expressions of C5aR1 in renal tubulointerstitia in DN patients were significantly higher than those of healthy controls (Figure 4A). In addition, compared with healthy controls, C5aR1 was mainly colocalized with and upregulated in macrophages in the renal tissues of DN patients, as revealed by immunofluorescence colocalization analyses (Figure 4B).

The WT and *C5aR1*^−/−^ mice were treated with HFD/STZ to investigate the function of native C5aR1 in DN. Weight loss, FPG and serum TCHO were significantly increased in the HFD/STZ-induced diabetic WT and *C5aR1*^−/−^ mice compared with the non-diabetic WT and *C5aR1*^−/−^ mice, respectively (Figure 4C). There were no significant changes in the weight loss, FPG and serum TCHO of the diabetic *C5aR1*^−/−^ mice compared with the diabetic WT mice (Figure 4C). Importantly, compared with the diabetic WT mice, the uACR was significantly decreased in the diabetic *C5aR1*^−/−^ mice (77.14 ± 29.30 vs. 318.50 ± 283.30 μg/mg, *p* = 0.0369) (Figure 4D), suggesting that the C5aR1 deficiency alleviated proteinuria independent of reducing obesity, hyperglycemia and hyperlipemia in the established DN mice. The PAS analysis showed significant improvements in the renal histological lesions in the diabetic *C5aR1*^−/−^ mice, including mesangial matrix accumulation and tubulointerstitial damage, in comparison to the diabetic WT mice (Figure 4E). The analysis using Sirius red staining revealed that the mice upon HFD/STZ treatment exhibited markedly enhanced accumulation of type I and III collagen in both the tubulointerstitial area and glomerular region. In contrast, the examination of renal tissue from the diabetic *C5aR1*^−/−^ mice demonstrated reduced staining compared with the diabetic WT mice, but it did not reach a statistical difference (Figure 4F).

### 3.5. Finerenone Alleviated HG-Induced Injuries and M1 Polarization of Macrophage via Inhibiting Gnαi2

**Figure 5:**
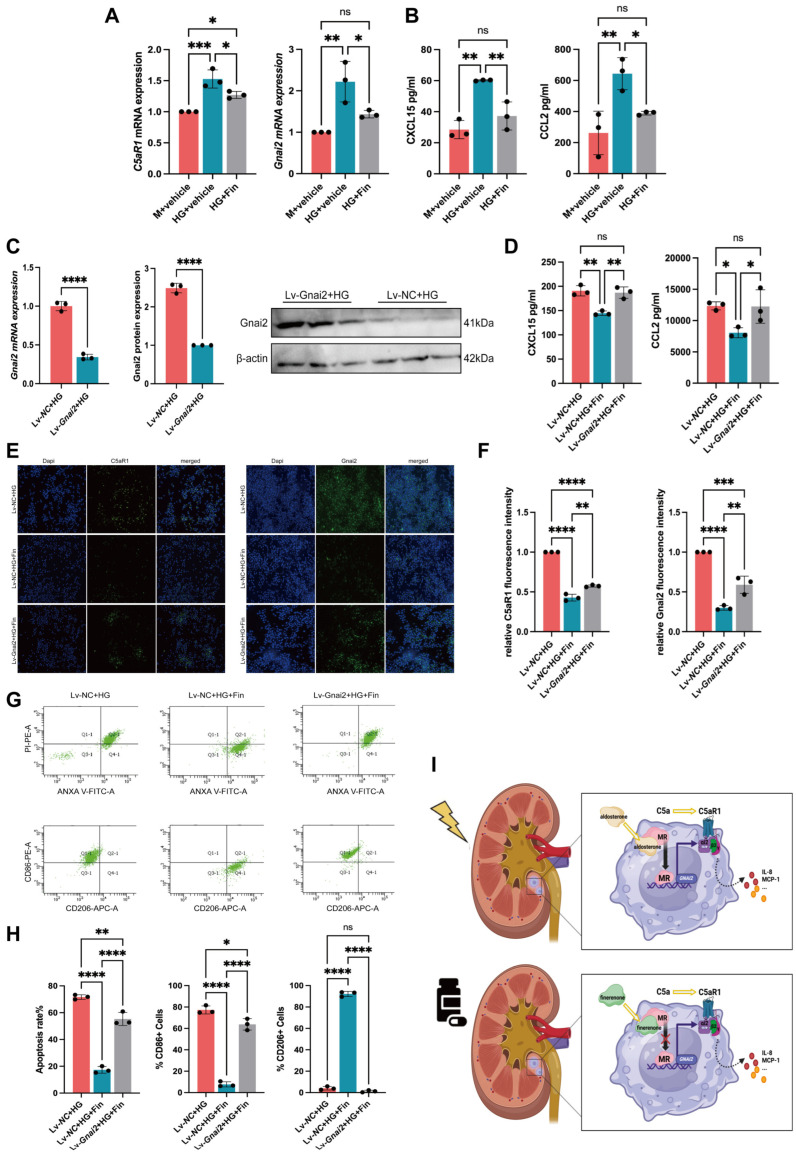
Finerenone alleviated HG-induced injuries and M1 polarization of macrophages via inhibiting Gnαi2. (**A**) mRNA levels of *C5aR1* and *Gnαi2* in three cell groups (*n* = 3 per group). (**B**) Levels of CXCL15 and CCL2 in cell supernatants of three groups (*n* = 3 per group). (**C**) Overexpression efficiencies of Gnαi2 were verified in both mRNA and protein levels (*n* = 3 per group). (**D**) Levels of CXCL15 and CCL2 in cell supernatants of three groups (*n* = 3 per group). (**E**) Representative cell IF images of C5aR1 and Gnαi2 in three cell groups (*n* = 3 per group). Scale bar: 50 μm. (**F**) Quantitative analysis of IF images of C5aR1 and Gnαi2 in three groups (*n* = 3 per group). (**G**) Representative images of Annexin V/PI, CD86 and CD206 by flow cytometry in three cell groups (*n* = 3 per group). (**H**) Proportions of apoptotic, M1 and M2 polarized cells in three groups were quantified, respectively (*n* = 3 per group). (**I**) Mechanism by which finerenone improved DN in this study. Asterisks Statistical significance levels are denoted as follows: * *p* < 0.05, ** *p* < 0.01, *** *p* < 0.001, **** *p* < 0.0001. The term ‘ns’ indicates a non-significant difference (*p* ≥ 0.05).

Consistent with the *in vivo* experiments, we found that compared with mannitol control, the mRNA expressions of *Ca5R1* and *Gnαi2* in RAW 264.7 macrophages were upregulated under high glucose conditions. Moreover, the levels of the chemokines CXCL15 and CCL2 in the cell supernatants were also upregulated, as determined by ELISA (Synergy H1, BioTek, Winooski, VT, USA). Meanwhile, the expressions of the above indicators were significantly downregulated after finerenone treatment in RAW 264.7 macrophages (Figure 5A,B). To confirm whether finerenone plays a role in regulating Gnαi2 expression, we overexpressed *Gnαi2* in the macrophages. Quantification of the mRNA and protein demonstrated effective enhancement of the expression (Figure 5C). Using ELISA, we found that the overexpression of *Gnαi2* abrogated the reduction of chemokines CXCL15 and CCL2 in the cell supernatants induced by finerenone under high glucose stimulation (Figure 5D). Immunofluorescence experiments showed that compared with the control group, the fluorescence intensity of Ca5R1 and Gnαi2 in RAW 264.7 macrophages were reduced upon finerenone treatment under high glucose stimulation, which could be increased by the overexpression of *Gnαi2* (Figure 5E). A semi-quantitative analysis of the immunofluorescence intensity in each group is shown in Figure 5F. Flow cytometry showed that finerenone treatment significantly reduced the proportions of apoptotic RAW 264.7 macrophages under high glucose. In contrast to the control group, the proportions were increased upon *Gnαi2* overexpression. In addition, we also found that compared with the control group, finerenone treatment reduced the proportions of M1 polarized macrophages, a pro-inflammatory phenotype marked by CD86, while it increased the M2 polarized macrophages, an anti-inflammatory phenotype indicated by CD206 under high glucose (M1: 7.733 ± 2.409 vs. 77.27 ± 3.765%, *p* < 0.0001; M2: 92.230 ± 2.376 vs. 4.067 ± 1.680%, *p* < 0.0001). However, the above improvement effects were abolished after *Gnαi2* overexpression, resulting in an increased M1/M2 ratio (mean ratio: 51.744 vs. 8.380, *p* < 0.0001) (Figure 5G). Figure 5H presents the results of the semi-quantitative analysis of the proportions of apoptotic and polarized macrophages in each group. The mechanism we demonstrated in this study is pictured in Figure 5I.

## 4. Discussion

Previous studies have reported that finerenone was considered a promising treatment of renal damage in DN through its anti-inflammatory and anti-fibrotic effects [9,10,11]. In our current study, we further explored the specific mechanism by which finerenone played the renoprotective role in DN, especially the effects on the complement C5a-C5aR1 axis and associated downstream pathways, particularly in macrophages.

Our results suggested that finerenone could improve DN by inhibiting the expression of Gnαi2, a potential key target of the MR. By inhibiting the expression of Gnαi2, finerenone alleviated the over-activation of the C5a-C5aR1 axis, which was known to play an important role in the pathogenesis of DN. C5aR1, primarily expressed in renal macrophages and renal tubular epithelial cells, was considered to participate in the progression of kidney injuries in DN through its recruitment of immune cells and chemokines and activation of inflammatory signaling pathways [17,19]. We found that both C5a-C5aR1-associated chemotaxis and kidney injury indicators were suppressed after the downregulation of Gnαi2 by finereone treatment in DN models. In addition, exploring the interactions between the C5a-C5aR1 axis and other inflammatory pathways such as TNF-α, IL-6 and NF-κB could reveal synergistic or compensatory mechanisms in DN. This could provide deeper insights into the complexity of inflammation and help develop more targeted therapeutic strategies. Future studies should focus on integrating these pathways for a better understanding of finereone treatment in DN.

This study also confirmed that complement components were essential mediators in DN progression. C5a, one of the most potent inflammatory mediators, was activated in DN and subsequently bound to C5aR1 to promote the recruitment of macrophages and the production of inflammatory cytokines and chemokines, contributing to further kidney injuries [12,24]. *In vivo*, we found that global knockout of *C5aR1* attenuated kidney injuries in HFD/STZ-induced diabetic mice, confirming the pathogenic role of endogenous C5aR1 in DN. By inhibiting the MR and subsequently downregulating the expression of Gnαi2, finerenone treatment significantly suppressed the over-activation of the complement C5a-C5aR1 axis and associated chemotaxis damages, thereby alleviating kidney damages in DN. These findings are consistent with previous studies in which the over-activation of the complement system correlated with the severity of DN progression.

Interestingly, our findings highlighted that finerenone has no direct impact on hyperglycemia or hyperlipidemia in DN, as evidenced by the significantly high fasting blood glucose and lipid levels in the finerenone treatment groups. However, its therapeutic effects appear to be more targeted to inflammation improvements, emphasizing its role as an immune response modulator rather than a metabolic modulator. This observation suggested that finerenone played an inflammatory and immune regulatory role in the treatment of DN, which is partially independent of metabolic control.

Moreover, the emerging role of the gut–kidney axis in DN warrants further discussion. The gut–kidney axis plays a significant role in DN progression, with gut dysbiosis contributing to systemic inflammation and renal injury through metabolites like short-chain fatty acids and uremic toxins [39]. While our study focused on the C5a-C5aR1 axis in macrophages, finerenone may indirectly influence the gut–kidney axis by reducing inflammation. Future studies incorporating microbiome analyses could uncover additional therapeutic insights.

Our study had limitations. Firstly, the established DN animal models and in vitro studies may not be able to fully replicate the situation of human DN pathologies. Further studies could be conducted to explore the clinical relevance by examining the impacts of the C5a-C5aR1-Gnαi2 axis, downstream inflammation and chemokines in DN patients who received regular oral finerenone therapy. Secondly, advanced glycation end products (AGEs) contribute to inflammation and fibrosis in DN [40]. Our bioinformatics analysis identified the C5aR1/Gnαi2 axis as the primary pathway, and thus we did not specifically investigate the impact of finerenone on AGE accumulation or associated pathways. Future studies integrating AGE measurements and their modulation by finerenone would provide a more comprehensive understanding of its therapeutic mechanisms. Thirdly, the 10-week treatment period in our study was unable to fully capture the long-term efficacy and safety of finerenone, warranting further studies to investigate chronic effects.

## 5. Conclusions

Taken together, we revealed the mechanism by which finerenone improved DN through modulating the complement system, specifically by inhibiting the C5a-C5aR1 axis via Gnαi2 downregulation in macrophages. Our findings provided new insight into the renoprotective role of finerenone and supported its potential as a promising strategy for DN.

## Figures and Tables

**Table 1:** Primer sequences used in real-time qPCR analysis.

Gene	Primer Sequence 5′ to 3′	
	Forward	Reverse
Mouse		
*C5ar1*	ATGGACCCCATAGATAACAGCA	GAGTAGATGATAAGGGCTGCAAC
*Gnαi2*	CAGAGGAACAAGGGATGCTTC	TAAGCGGCTGAGTCATTGAGC
*Cxcl15*	CAAGGCTGGTCCATGCTCC	TGCTATCACTTCCTTTCTGTTGC
*Ccl2*	TTAAAAACCTGGATCGGAACCAA	GCATTAGCTTCAGATTTACGGGT
*18 s*	GTAACCCGTTGAACCCCATTC	GCCTCACTAAACCATCCAATCG

## Data Availability

The datasets presented in this study could be found in online repositories. The names of the repository could be found in the article. The data used to support the findings of this study are available from the corresponding author upon request.
